# Microbial Diversity and Putative Opportunistic Pathogens in Dishwasher Biofilm Communities

**DOI:** 10.1128/AEM.02755-17

**Published:** 2018-02-14

**Authors:** Prem Krishnan Raghupathi, Jerneja Zupančič, Asker Daniel Brejnrod, Samuel Jacquiod, Kurt Houf, Mette Burmølle, Nina Gunde-Cimerman, Søren J. Sørensen

**Affiliations:** a Molecular Microbial Ecology Group, Section of Microbiology, Department of Biology, University of Copenhagen, Copenhagen, Denmark; b Department of Biology, Biotechnical Faculty, University of Ljubljana, Ljubljana, Slovenia; c Department of Veterinary Public Health and Food Safety, Faculty of Veterinary Medicine, Ghent University, Ghent, Belgium; The Pennsylvania State University

**Keywords:** abiotic conditions, biofilms, dishwasher, fungal-bacterial interactions, opportunistic fungi, putative pathogens

## Abstract

Extreme habitats are not only limited to natural environments, but also exist in manmade systems, for instance, household appliances such as dishwashers. Limiting factors, such as high temperatures, high and low pHs, high NaCl concentrations, presence of detergents, and shear force from water during washing cycles, define microbial survival in this extreme system. Fungal and bacterial diversity in biofilms isolated from rubber seals of 24 different household dishwashers was investigated using next-generation sequencing. Bacterial genera such as Pseudomonas, Escherichia, and Acinetobacter, known to include opportunistic pathogens, were represented in most samples. The most frequently encountered fungal genera in these samples belonged to Candida, Cryptococcus, and Rhodotorula, also known to include opportunistic pathogenic representatives. This study showed how specific conditions of the dishwashers impact the abundance of microbial groups and investigated the interkingdom and intrakingdom interactions that shape these biofilms. The age, usage frequency, and hardness of incoming tap water of dishwashers had significant impact on bacterial and fungal community compositions. Representatives of Candida spp. were found at the highest prevalence (100%) in all dishwashers and are assumed to be one of the first colonizers in recently purchased dishwashers. Pairwise correlations in tested microbiomes showed that certain bacterial groups cooccur, as did the fungal groups. In mixed bacterial-fungal biofilms, early adhesion, contact, and interactions were vital in the process of biofilm formation, where mixed complexes of bacteria and fungi could provide a preliminary biogenic structure for the establishment of these biofilms.

**IMPORTANCE** Worldwide demand for household appliances, such as dishwashers and washing machines, is increasing, as is the number of immunocompromised individuals. The harsh conditions in household dishwashers should prevent the growth of most microorganisms. However, our research shows that persisting polyextremotolerant groups of microorganisms in household appliances are well established under these unfavorable conditions and supported by the biofilm mode of growth. The significance of our research is in identifying the microbial composition of biofilms formed on dishwasher rubber seals, how diverse abiotic conditions affect microbiota, and which key microbial members were represented in early colonization and contamination of dishwashers, as these appliances can present a source of domestic cross-contamination that leads to broader medical impacts.

## INTRODUCTION

Extreme natural environments have for decades attracted the interest of microbiologists. However, microbial communities in extreme environments in households and common household appliances have only been studied fairly recently. The selection pressure within some of these environments is reflected in reduced microbial diversity, allowing only the most fit species to withstand the stressful conditions. In recent years, in addition to understanding the biodiversity of extreme natural habitats (1–5), ecological investigations into various manmade ecosystems, such as kitchens (6), bathrooms (7), trash bins (8), tap water pipes (9, 10; see also M. N. Babič, P. Zalar, and N. Gunde-Cimerman, presented at ISHAM, Guangzhou, China, 2013), automated teller machines (11), coffee machines (12), washing machines (13, 14), and dishwashers (15–18), have gained momentum.

Among the different household ecosystems studied so far, kitchens are colonized by the broadest diversity of extremotolerant microorganisms (19–21). Only recently, it was discovered that some microbes can survive and grow even under extreme conditions in certain domestic appliances (12, 15) like, for instance, dishwashers (DWs). DWs are extreme habitats with constantly fluctuating conditions, where only microbial communities with polyextremotolerant properties can survive. The individual community members, as well as the whole community itself, must possess key phenotypic traits that enable them to resist alternating wet and dry periods, frequent changes of temperature during the washing cycles (from 20°C to 74°C), oxidative detergents that elevate the pH from 6.5 to 12, high organic loads, high NaCl concentrations, and shearing generated by water sprinklers. The metal, plastic, and rubber parts of DWs may enable the establishment and growth of mixed bacterial-fungal communities that are protected by copious amounts of extracellular polymeric substances (EPSs), which confer on the biofilm communities extremotolerant properties that go beyond the extremotolerance of each individual species (16, 17, 22).

The obvious choice for microbes when exposed to extreme conditions in DWs is the biofilm mode of growth, which provides shelter against external stresses (23) and where intimate cross-species boundaries may occur (24). Such surface communities (25, 26) may also provide a link to emerging disease pathogenesis (27), since biofilms formed in DWs (and other appliances) could contribute to the dispersion and persistence of bacterial-fungal groups outside the common spectrum of saprobes (15). Fungi able to cause opportunistic infections in humans have been documented inside DWs (16–18), and the incidence of domestically sourced fungal infections has been increasing steadily in recent decades (16, 28, 29).

However, to date, the diversity of the whole microbiota in DWs has not been investigated. Therefore, we have studied both the bacterial and fungal communities (with a special focus on mixed biofilms) associated with household DWs, using high-throughput sequencing. Furthermore, we studied how specific conditions of the DWs impact the abundance of certain microbial groups and how inter- and intrakingdom interactions shape the structure of microbial communities within DWs.

## RESULTS

### Characterization of bacterial and fungal communities in DW-associated biofilms.

**FIG 1:**
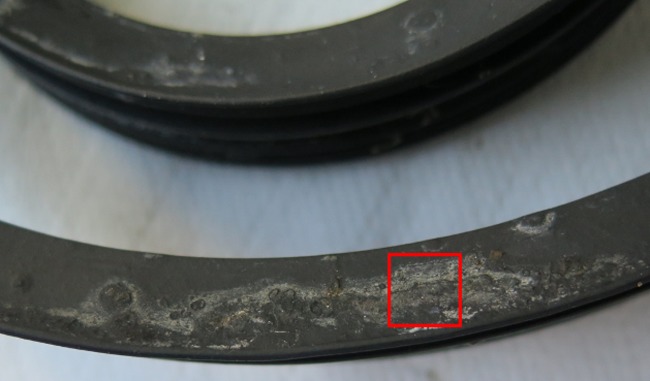
Biofilm formed on rubber seals in residential DWs. Microbial biofilm formation on DW rubber seal; the square (in red) represents the 1-cm^2^ sampling site. Biofilm samples from 1 cm^2^ were collected by scraping the surface of rubber seal with a sterile scalpel for DNA extraction and further analysis. Sampling was done *in situ*, with the seal at its original place.

Twenty-four biofilms grown on rubber seals of DWs (Fig. 1) were sampled to assess both bacterial and fungal communities. DW characteristics varied in terms of years of use, frequency of use, temperature of washing cycles, and influent water hardness (WH), as shown in Table 1. In the first PCRs, 21 samples generated DNA fragments that were processed by a second PCR and further sequenced. A total of 221,032 partial 16S rRNA gene and 313,420 internal transcribed spacer (ITS) rRNA gene transcript sequences were obtained from 21 DW samples. Raw sequences of all the DW samples are available from the NCBI Sequence Read Archive (SRA) under the BioProject accession numbers PRJNA315977 for bacterial reads and PRJNA317625 for fungal reads. Overall, sequence reads were assigned to 309 bacterial operational taxonomic units (OTUs) and 194 unique fungal OTU classifications. The predicted number of bacterial genera ranged from 29 to 150 across all samples and that of the fungal genera ranged from 15 to 104 (see Table S1 in the supplemental material).

**TABLE 1:** Different dishwasher machines and their associated characteristics from which biofilm samples were obtained^*a*^

Sampled dishwasher^*a*^	Partial sequence obtained for^*b*^:		Age (yrs used)	Frequency of use (no. of times/wk)	Washing cycle temp (°C)	Water hardness^*c*^
	16S rRNA gene	ITS rRNA gene				
S1	✓	✓	2.5	7	60	SH
S2	✓	✓	2.5	7	60	SH
S3	✓	✓	7	3	60	MH
S4	✓	✓	3	14	60	MH
S5	✓		2	14	60	MH
S6	✓	✓	8	7	60	MH
S7	✓	✓	5	3	60	MH
S8		✓	8	7	60	MH
S9		✓	5	3	60	MH
S10	✓		1	2	65	MH
S11	✓	✓	7	7	60	SH
S12	✓	✓	0.5	3	65	SH
S13	✓	✓	1	3	50	SH
S14	✓		2	2	65	SH
S15	✓	✓	8	7	65	MS
S16	✓	✓	1	7	65	MH
S17	✓	✓	1	14	65	MH
S18		✓	4	3	65	MH
S19	✓	✓	4	3	65	H
S20	✓	✓	3	1	60	SH
S21	✓	✓	8	7	50	MS
S22	✓	✓	5	7	60	H
S23	✓	✓	1	1	65	SH
S24	✓	✓	8	7	60	MS

a *n* = 24.

b Check marks (✓) represent the samples that were sequenced based on 16S rRNA genes/ITS rRNA region.

c H, hard (more than 2.0 mmol/liter CaCO_3_); MH, moderately hard (1.5 to 2 mmol/liter CaCO_3_); SH, slightly hard (1.0 to 1.5 mmol/liter CaCO_3_); MS, moderately soft (0.5 to 1 mmol/liter CaCO_3_); S, soft (less than 0.5 mmol/liter CaCO_3_).

Alpha diversity indices were calculated based on rarefied sequences and are presented (see Table S2 in the supplemental material). Richness and evenness of the bacterial communities were not affected by the DW conditions (see Table S3 in the supplemental material). However, the alpha diversity indices of the fungal communities were found to be significantly influenced by DW conditions. The years of usage of the DW and presence of influent hard water had significant impact on species richness, species evenness, and abundance, indicating that these specific environmental conditions enrich fungal community profiles (Wilcoxon-Mann-Whitney test, *P* < 0.05) (see Table S4 in the supplemental material).

### Microbial phyla in DW-associated biofilms.

**FIG 2:**
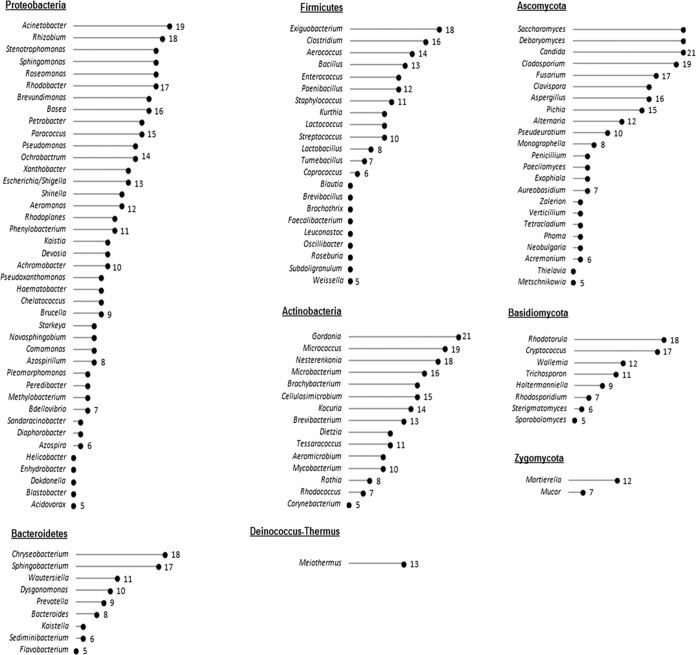
The different bacterial and fungal genera are presented across 21 DW samples. The numbers represent the sample count, i.e., the number of DW samples that contained the representative genera.

DW biofilms were composed of diverse fungal and bacterial phyla. Based on nonrarefied 16S rRNA gene reads, the sequences were assigned to 16 different bacterial phyla, of which Proteobacteria dominated across all samples, followed by Actinobacteria and Firmicutes (see Fig. S1 in the supplemental material). All DW samples also contained sequences representing members of the Bacteroidetes. The remaining phyla (Chloroflexi, Cyanobacteria, Deinococcus-Thermus, Spirochaetes, TM7, Verrucomicrobia, Synergistetes, and Acidobacteria) contributed up to 9% of the total prokaryotic sequences. Among the subclasses of Proteobacteria, the bacterial community was dominated by Alphaproteobacteria (46% ± 7%) and Gammaproteobacteria (45% ± 7%) in all of the DW biofilm samples. Bacilli was the most abundant subclass of Firmicutes, dominating in all biofilm samples. The most abundant taxa in all DNA samples belonged to the genera Gordonia, Wautersiella, Rhodobacter, Nesterenkonia, Stenotrophomonas, Exiguobacterium, Acinetobacter, and Pseudomonas. Bacterial OTUs represented by the genus Meiothermus belonging to the phyla Deinococcus-Thermus and TM7 were present in 19/21 DW samples. The genera Escherichia/Shigella and Pseudomonas were identified in 62% and 67% of DWs, respectively (Fig. 2).

OTUs based on nonrarefied ITS rRNA gene reads were assigned across four fungal phyla (Fig. S1). The Ascomycota dominated in the samples, followed by the Basidiomycota. Among the subclasses of Ascomycota, the fungal biofilm community was dominated by Saccharomycetes, which was characterized in all DWs by the genera Candida, Debaryomyces, and Saccharomyces (Fig. 2). Filamentous fungal genera like Cladosporium, Fusarium, and Aspergillus were present in more than 16 DW samples, and the black yeast genera Aureobasidium and Exophiala were present in 33% of DWs. Members of the phylum Basidiomycota, represented by the genera Rhodotorula and Cryptococcus, were present in 90% and 86% of DWs, respectively. The genera Wallemia and Trichosporon were present in more than half of DWs. The microbiota based on absolute sample count, i.e., the bacterial and fungal taxa classified at the genus level that occurred in more than 5 samples, where 21 ≥ *n* ≥ 5, is shown in Fig. 2.

### Abiotic conditions of DWs affect microbial composition.

**FIG 3:**
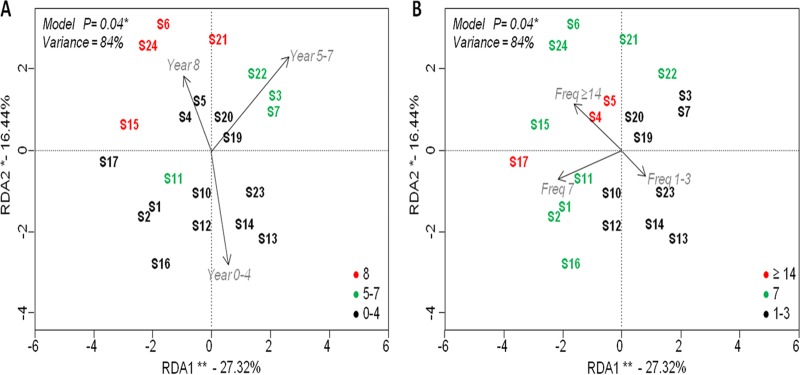
Principal component plots of redundancy analysis (RDA) performed on log_10_-transformed 16S rRNA amplicon sequencing data using years of use (A) and frequency of use (B) as explanatory factors. Significance of the model, axes, and factors was determined by ANOVA (999 permutations; *P* < 0.05). Asterisks stand for the level of significance as follows: *, 0.05 ≤ *P* < 0.01; **, 0.01 ≤ *P* < 0.001. Year, years of use (0 to 3, 5 to 7, and 8 years); Freq, frequency of use (1 to 3, 7, and ≥14 times/week).

**FIG 4:**
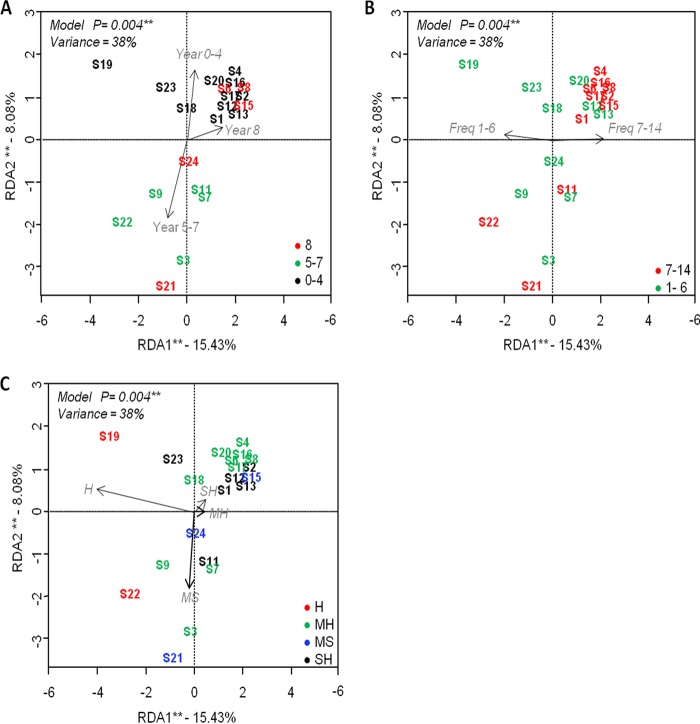
Principal component plots of redundancy analysis (RDA) performed on log_10_-transformed ITS rRNA amplicon sequencing data, using years of use (A), frequency of use (B), and water hardness (C) as explanatory factors. Significance of the model, axes, and factors was determined by ANOVA (999 permutations; *P* < 0.05). Asterisks stand for the level of significance as follows: *, 0.05 ≤ *P* < 0.01; **, 0.01 ≤ *P* < 0.001. Year, years of use (0 to 3, 5 to 7, and 8 years); Freq, frequency of use (1 to 3, 7 and ≥14 times/week); H, hard water; MH, moderately hard water; SH, slightly hard water; MS, moderately soft water.

Changes in the structure of microbial communities were investigated by multivariate beta diversity analysis. Redundancy analyses (RDAs) were performed to test the relationships between different DW conditions and their impacts on the microbial community composition. RDA with two included factors (years of use and frequency of use) showed that the DW years of use was the most significant driving force (analysis of variance [ANOVA], *P* < 0.05) affecting bacterial communities, followed by frequency of use (Freq). RDA explained 84% of the total variance, of which 43.8% was described by RDA1 and RDA2 (27.32% and 16.44%, respectively; Fig. 3A and B). The most explanatory variables were frequency of use (27%) and the number of years in use (23%) (see Table S5 in the supplemental material). RDA with three included factors (year, water hardness, and frequency of use) showed that years of use, frequency of use, and WH significantly affected the fungal community (ANOVA, *P* < 0.05). Out of 38% total variance, 23.5% was explained by the first two components (15.43% and 8.08%, respectively; Fig. 4A, B, and C). In this case, WH was found to be the most explanatory variable (18.9%) (see Table S5). Permutational analysis of variance (PERMANOVA) on the Bray-Curtis dissimilarity index confirmed the observed trends, where DW frequency of use (14%) and WH (17%) were the most significant factors (*P* < 0.05) impacting the bacterial and fungal community profiles, respectively (see Table S6 in the supplemental material).

Redundancy analysis (RD) scatterplotting shows DWs with bacterial composition grouped into recently purchased DWs used for 0 to 4 years and old DWs at 5 to 7 or 8 years of use (Fig. 3A). Based on frequency of use, DWs were grouped into three categories: low-frequency (1 to 3 times/week), intermediate-frequency (7 times/week) and high-frequency (14 times/week) use (Fig. 3B). Samples with fungal composition were also grouped based on DW conditions (year, frequency of use, and influent WH) as shown in Fig. 4A, B, and C, respectively.

### Influence of DW age and frequency of use on bacterial communities.

**FIG 5:**
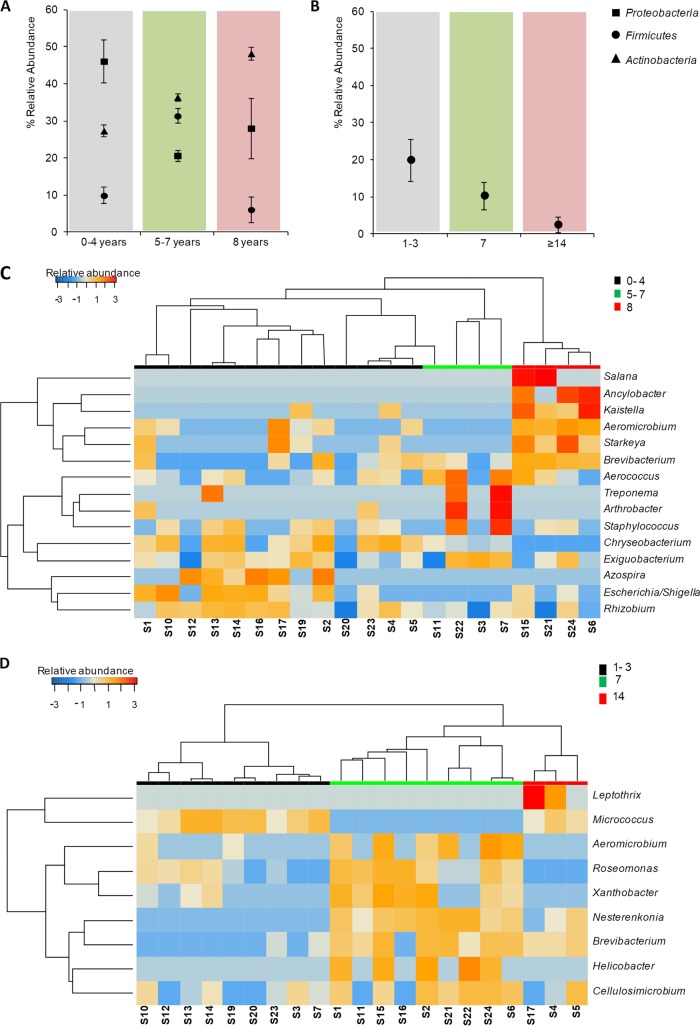
Impact of age and frequency of use of DWs on the relative abundance of bacterial taxa present in the samples. (A and B) Mean ± standard error of the mean (SEM) of percent abundance in samples grouped by years of use (A) and frequency of use (B) at the phylum level. (C and D) Heatmap of significant bacterial genera in DW samples grouped by years of use (0 to 4, 5 to 7, and 8 years) (C) and frequency of use (1 to 3, 7, and ≥14 times/week) (D), respectively.

Based on the above results, further analyses of DW groups by taxonomic profile were performed in the software package STAMP. Actinobacteria, Firmicutes, and Proteobacteria were the three major phyla significantly affected by DW age (ANOVA, *P* < 0.05), and the phylum Firmicutes was influenced further by frequency of use (ANOVA, *P* < 0.05). Recently purchased DWs (0 to 4 years old) contained Proteobacteria (48%), Actinobacteria (29%), and Firmicutes (13%), indicating that members of these phyla were early settlers in biofilms of recently purchased DWs. DWs between 5 and 7 years of use seem to be increasingly populated with age by Actinobacteria (36%) and Firmicutes (34%). DWs at 8 years of use showed increased levels of Actinobacteria (49%), with further reduction in levels of Proteobacteria and Firmicutes (Fig. 5A). DWs that were used more often (frequency) had reductional shifts in Firmicutes diversity (Fig. 5B). Thus, bacterial members belonging to the groups Actinobacteria and Proteobacteria dominated in DWs, with Firmicutes being susceptible to operational conditions of DWs.

Investigation of abundance shift patterns at genus level between different DW age groups is shown in Fig. 5C. A total of 11 bacterial taxa showed significant levels of prevalence in the three DW age groups (ANOVA, *P* < 0.05). Most young DWs (0 to 4 years) had various levels of bacterial abundance, represented by the genera Rhizobium, Escherichia/Shigella, Azospira, Exiguobacterium, Chryseobacterium, and Staphylococcus. Taxa belonging to the genera Exiguobacterium, Arthrobacter, Staphylococcus, Aerococcus, Treponema, and Lactobacillus were represented in DWs at 5 to 7 years of use. Furthermore, genera such as Aeromicrobium, Salana, Ancylobacter, Starkeya, Kaistella, Brevibacterium, and Ancylobacter dominated in DWs used for 8 years. The genera Azospira, Escherichia/Shigella, and Rhizobium dominated in younger DWs (Welch's *t* test, *P* < 0.05), whereas the genera Aeromicrobium and Brevibacterium dominated in older DWs used for 5 to 7 or 8 years (Welch's *t* test, *P* < 0.05, respectively). The frequency of use analysis showed prevalences of 9 bacterial taxa to be significantly influenced (ANOVA, *P* < 0.05) among the three levels (Fig. 5D). The genera Brevibacterium, Aeromicrobium, Roseomonas, Xanthobacter, Helicobacter, and Cellulosimicrobium were prevalent in DWs used more frequently compared to DWs used at low frequencies (1 to 3 times/week) (Welch's *t* test, *P* < 0.05).

### Fungal community of DW rubber seals is influenced by age, frequency of use, and hardness of incoming tap water.

**FIG 6:**
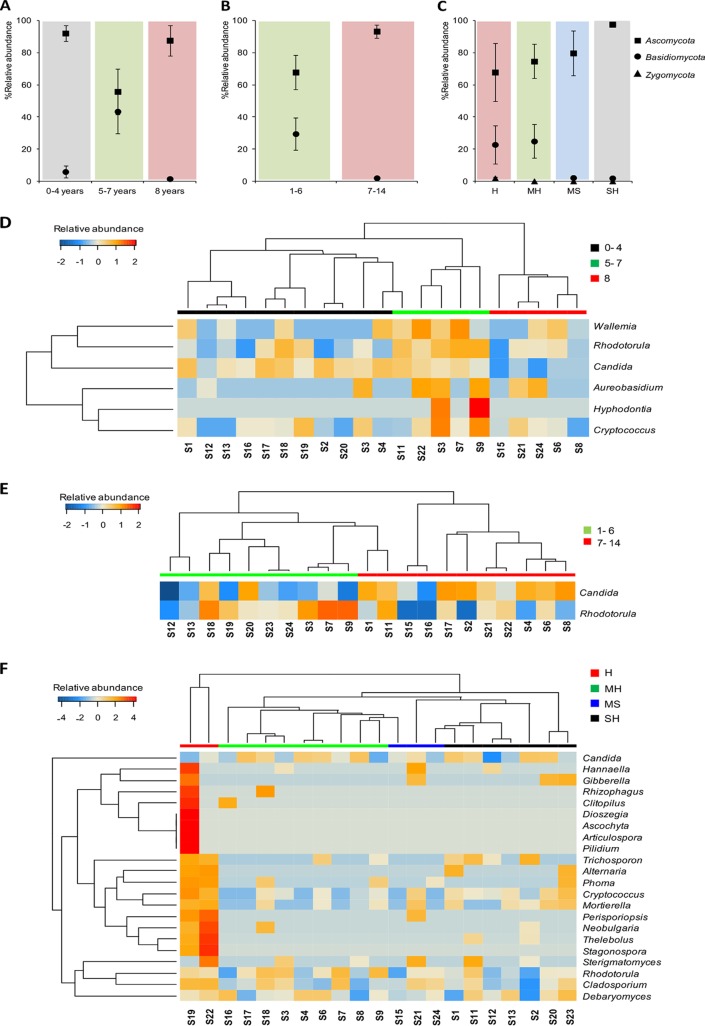
Impact of DW age and frequency of use on the relative abundance of fungal taxa present in the samples. (A and B) Mean ± SEM of percent abundance in samples grouped by years of use (A), frequency of use (B), and water hardness (C) at the phylum level. (D, E, and F) Heatmap of significant fungal genera in DW samples grouped by years of use (0 to 4, 5 to 7, and 8 years) (D), frequency of use (E), and influent water hardness (F), respectively. H, hard water; MH, moderately hard water; SH, slightly hard water; MS, moderately soft water.

The conditions of DWs and their impacts on fungal taxonomic profiles revealed a significant difference between the two fungal phyla Ascomycota and Basidiomycota (ANOVA, *P* < 0.05). Young dishwashers (0 to 4 years of use) showed abundant levels of fungi from the phylum Ascomycota (92%), with Basidiomycota contributing only 5%. Over time, the two phyla became more equally represented at 55% and 43%, respectively (Fig. 6A). A larger shift in abundance between the phyla Ascomycota and Basidiomycota was observed under two use frequency levels (Welch's *t* test, *P* < 0.05, respectively) (Fig. 6B). This could indicate that Basidiomycota genera are susceptible to decline in DWs used more frequently, while ascomycetous fungi remain well established. All levels of tap water hardness were shown to affect the phyla Ascomycota, Basidiomycota, and Zygomycota (ANOVA, *P* < 0.05). Hard and moderately hard water influenced fungal genera belonging to Basidiomycota and Zygomycota compared to soft and slightly hard water (Welch's *t* tests, *P* < 0.05) (Fig. 6C).

At genus level, abundance levels of 6 taxa were influenced by years of use (Fig. 6D). Most DWs used for between 5 and 7 years were shown to be colonized by the genera Wallemia, Rhodotorula, Candida, Aureobasidium, and Cryptococcus, although recently purchased DWs had various fungal representations among these genera. Candida was significantly abundant in recently purchased DWs (0 to 4 years of use), and Rhodotorula was significantly higher in abundance in DWs used for 5 to 7 years compared to DWs used for 8 years (Welch's *t* test, *P* < 0.05), respectively. DW frequency of use had significant impact on the fungal genera Candida and Rhodotorula, whereas most frequently used DWs were enriched in Candida and less frequently used DWs were settled with Rhodotorula (Welch's *t* test, *P* < 0.05) (Fig. 6E). The incoming WH also influenced the fungal biota, as shown in Fig. 6F. The genera Phoma, Thelebolus, Stagonaspora, Neobulgaria, Perisporiopsis, and Cladosporium were represented at significantly higher abundance in samples with hard water than in samples with other WH characteristics (Welch's *t* test, *P* < 0.05).

### Positive microbial interactions may shape DW biofilm communities.

Microbes sharing the same environmental niche may coexist or exclude each other while competing for the same resources (30–32). Correlation analyses were performed to explore relationships among the microbial flora associated with DW biofilm communities, as positive pairwise correlations may indicate microbial collaboration or dependencies. The survey revealed 140 significant interactions at the genus level in biofilm samples, including both fungal and bacterial genera (Spearman's rank correlation coefficient cutoff, *r* > ∣0.65∣; permutation test, *P* < 0.05), with 90% (125/140) of the predicted interactions positively correlated (Table 2). Surprisingly, 95% (119/125) of the predicted positive correlations were intrakingdom bacterial interactions dominated by Proteobacteria, Actinobacteria, and Firmicutes. Representatives of genera detected in these biofilms were shown to positively correlate with presence of other taxa. For example, the presence of Gammaproteobacteria genus Escherichia/Shigella spp. was positively correlated with that of Ochrobactrum spp., Staphylococcus spp. was positively correlated with the Gammaproteobacteria genus Pseudomonas, and Enterococcus spp. was positively correlated with the Gammaproteobacteria genus Stenotrophomonas. Positive interkingdom correlations, i.e., interactions between bacterial and fungal members, were observed only in 2.5% of total positive interactions (*n* = 125). Examples include interactions between Saccharomycetes fungi (Candida spp.) and Alphaproteobacteria in one case and between Betaproteobacteria and Dothideomycetes fungi in two cases. Fungal members tend to mutually cooccur in DW communities (2.5% [3/125]), as no negative correlations within the fungal taxa were observed. In fungi, positive cooccurrence was observed between Rhodotorula spp. and Cladosporium spp., Cryptococcus spp. and Cladosporium spp., and Debaryomyces spp. and yeasts classified as Saccharomycetes. However, mutual exclusions among interkingdom interactions accounted for 46% (7/15) of the total negative correlations (*n* = 15). Cross-domain negative interactions, i.e., mutual exclusions between bacteria and fungi, were observed between the fungal phylum Ascomycota and the bacterial phyla Actinobacteria and Bacteroidetes. Basidiomycota fungi negatively correlated with Gammaproteobacteria, and Alphaproteobacteria negatively correlated with Sphingobacteria (see Fig. S2 in the supplemental material).

**TABLE 2:**
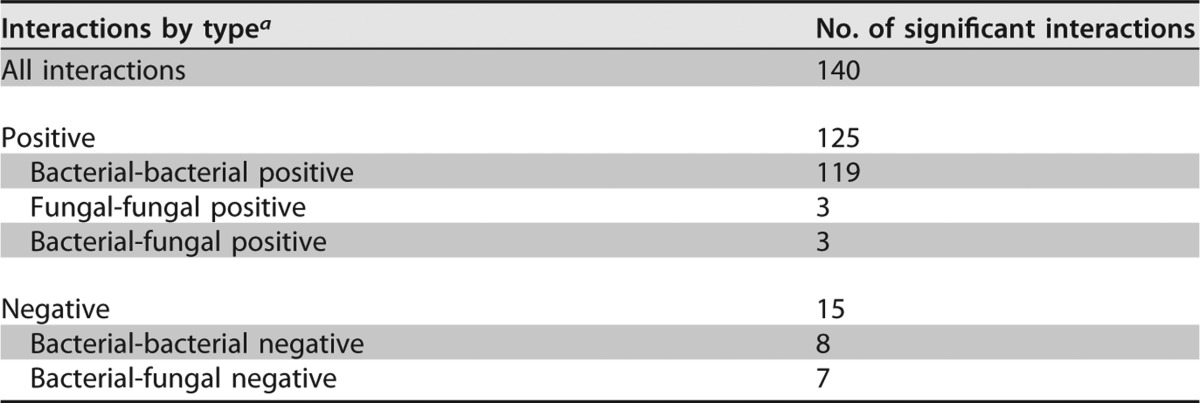
Pairwise correlations between bacterial and fungal OTUs observed in 18 DW biofilm samples

*^a^* Interactions shown here are based on Spearman's rank correlation coefficients (*r* > cutoff = |0.65|; permutation test, *P* < 0.05).

## DISCUSSION

Indoor and household environments, including those of household appliances, offer diverse habitats for microorganisms to adapt and flourish. Most current knowledge of DW microbiology is focused on opportunistic pathogenic black yeasts and other members of DW mycobiota characterized based on classical cultivation techniques (15, 16, 18). To date, the molecular approach to identifying and characterizing the microbial diversity of DWs was documented in a single study that was limited to the presence of fungi in new and old biofilms (16). This study, using high-throughput sequencing, assessed both bacterial and fungal community coexistence in biofilms that were developed on the rubber seals of DWs. These mixed bacterial-fungal biofilms are very likely to enable protection against harsh environmental conditions, contributing to persistence. The formation of biofilms on DW rubber seals also reflects invasion, settlement, and growth of microorganisms under extreme conditions in these appliances.

A combination of different stress factors within DWs allows for the survival of only well-adapted and complementary microbial species that enable the formation of biofilms. We found that on rubber seals in DWs, most abundant OTUs were dominated by Gram-positive members like Gordonia spp., Micrococcus spp., Chryseobacterium spp., and Exiguobacterium spp. The presence of these genera is usually associated with natural environments in which biotic conditions are extreme (33–38). A few species within these genera were earlier reported as halotolerant and tolerant of a broad range of pHs (5–10; see also M. N. Babič, P. Zalar, and N. Gunde-Cimerman, presented at ISHAM, Guangzhou, China, 2013), high levels of UV radiation, and heavy metal stress (including arsenic) (33–35). The most abundant bacterial genus, Gordonia, was found in all DWs sampled in this study. These representatives were reported to degrade different polymers and xenobiotics (37) that presumably facilitate their presence on DW rubber seals. Bacterial taxa represented by the putative thermophilic genus Meiothermus and by phylum TM7 were present in most DW samples. Both of these thermophilic genera could be expected in biofilms formed on DW rubber seals, since the bacterial members of these genera can tolerate short periods of up to 70°C and grow optimally from 50 to 65°C and under alkaline conditions (pH ∼8.0) (38). This study reports the detection of putative Meiothermus spp. within a domestic system.

Microbial diversity within indoor environments is influenced by human effects. The abundances in bacterial composition of an indoor environment were shown to closely mirror the microbial profiles of its human residents (39). Sampled DWs included a subset of bacterial genera known to have representatives associated with humans, for example, Staphylococcus, Streptococcus, Lactobacillus, Corynebacterium, and Enterococcus. These bacterial genera, common on human skin and in the gut, have been detected in other studies investigating the domestic microbiome (29, 40, 41), yet their presence under extreme conditions has not been reported. Furthermore, our study revealed the presence of gene sequences affiliated with genera known to harbor some of the most common and potential human opportunistic pathogens, namely, Escherichia/Shigella and Pseudomonas, as integral members of DW microbial biofilms. In fact, more than 60% of samples contained genera such as Acinetobacter, Escherichia/Shigella, and Pseudomonas, indicating that DWs could shelter these bacterial groups in private homes. However, it should be noted that the presence of potential bacterial pathogens in household DWs can be tempered by the fact that pathogenicity can be strain specific (the methods used here do not provide such resolution). In addition, the approach applied in our study cannot distinguish between alive/dead cells and spores. Multispecies biofilm formation may help these well-adapted (and other) opportunistic bacteria to survive in harsh environment of DWs, being protected in the immediate vicinity with large amounts of bacterial and fungal EPSs (30, 31). EPSs immobilize individual cells in biofilms and keep them in close proximity, allowing interactions, including cell-cell communication and the formation of synergistic microconsortia (30), which thereby determine the development and structure of multispecies biofilms (32). The primary colonizers of any surface are predominantly bacteria (42), which modify surface characteristics, enabling subsequent colonization by secondary microorganisms (43, 44). Recent studies of microbial biofilm communities on natural and artificial substrates (ceramic, glass, plastic, aluminum, and coral skeleton) showed that early stage biofilms were established by successive colonization of Proteobacteria, Firmicutes, and Actinobacteria (45–47). Similarly, in this study, it was observed that in recently purchased dishwashers, the main biofilm community members belonged to the phyla Proteobacteria, Actinobacteria, and Firmicutes. Alphaproteobacteria that prevail in aquatic-related biofilms (48), in addition to gammaproteobacteria, were well represented in DWs. Gammaproteobacteria were found to be the major contributors to biofouling (48, 49) formed on polymers (50), and their presence in DWs could correlate to different polymers used in DW components.

Taxonomic identification of the fungal DW community by gene marker-based amplicon sequencing showed results similar to those of previous cultivation-based approaches (15, 16, 18). The prevalence of Ascomycota in relation to Basidiomycota was confirmed (16). However, in this study, we report differences in distribution at the genus level. Sequencing results from fungi in DW biofilms from a previous study showed a higher abundance of Cryptococcus (16), while in this study the dominance of Candida is reported. Also, the prevalence of fungi belonging to the genera Rhodotorula and Cryptococcus was higher, and the genera Exophiala and Aureobasidium, the two black yeast-like fungi classified as opportunistic pathogens, were also detected. The presence of black yeast-like fungi was lower than that in previous cultivation-based studies (15–18). This reduced incidence rate of these colonizers in DWs could be the consequence of less efficient DNA extraction from black yeast cells due to the presence of melanin in cell walls, large polysaccharide production, or meristematic growth forms (51–53). In addition, lower or higher incidence of microbial composition can also result from the chosen methodology, as sequencing analyses will also detect nonviable cells (54), resulting in increased microbial richness.

The density of microbial settlement on 1-cm^2^ rubber seals in DWs is only known for the fungal community (16) and not for the bacterial community. The main colonizer black yeast, E. dermatitidis, was detected at up to 10^6^ CFU/cm^2^; E. phaeomuriformis, R. mucilaginosa, and C. parapsilosis were detected in the range of 10^2^ to 10^5^ CFU/cm^2^ (16). Ascomycetous fungi, namely Candida, dominated the recently purchased DWs. These early fungal colonizers were followed by other cooccurring Ascomycota and Basidiomycota members. It cannot be excluded that early colonizers may benefit from the so-called “priority effect” (55), giving them the advantage of occupying the surface first, and subsequently filtering/choosing the newcomers, resulting in differential community assemblies. The hardness of tap water also significantly affected the fungal community in DWs, such that more diverse fungal species were present in DWs with hard and moderately hard water. Hard water and moderately hard water have increasing contents of Ca^2+^ and Mg^2+^ ions. Although water disinfection procedures have also been shown to influence fungal diversity (56, 57), it is noteworthy that the existence and morphology of certain fungi depend on the presence of some ions, in particular cations such as Ca^2+^ (58). Other ions, such as Cl^−^, generated by water chlorination, generally do not affect fungi (59, 60). Our results indicate that establishment and diversity of fungi within DW biofilms will be greater with hard water, while in soft water fungal biomass tends to be dominated by the fungal phylum Ascomycota. Furthermore, fungal composition could also be influenced by adaption to different disinfectants used during wash cycles in DWs.

In most natural environments, individual organisms do not live in isolation but rather form a complex community of different species that shape the structure of the community itself and the evolution of the individual species (61). In mixed bacterial-fungal biofilms, early contact and adhesion are likely to be important in the process of biofilm formation, where bacteria, fungi, or mixed complexes of the two might provide biotic support for the establishment of biofilms (62–64). Microbes that live in the same ecological habitat may cooccur or exclude each other. Studies have shown that coexistence can facilitate interspecies interactions in biofilms (31, 65). This aspect was investigated, as the fungal and bacterial communities from 18 samples in this study shared the same habitat and therefore could provide insights into possible interactions. Positive pairwise correlations indicate mutual cooccurrence, which also may point to symbiosis, mutualism, or commensalism, whereas negative pairwise correlations indicate mutual exclusion, which may reflect competition, mutual exclusion, or parasitism (66). Our results indicate that bacterial groups cooccur with each other, as do the fungal groups with other fungal members. Interestingly, cross-kingdom pairwise correlations between fungi and bacteria were dominated by negative correlations, which may reflect occupation of different locations on the biofilms.

However, *in vitro* studies have shown close interactions between yeast cells forming the biofilm core and bacteria in the biofilm periphery that create a protective coating for yeasts cells and pseudohyphae (67, 68). Stressful conditions in DWs and the presence of bacteria could stimulate growth of fungi like Candida spp. as pseudohyphae, thus enabling formation of the multispecies biofilm core on which further bacteria could associate. This could be attributed to the positive cross-kingdom correlation seen between Saccharomycetes (mostly represented by the genus Candida) and Proteobacteria (Alphaproteobacteria). This suggests that the early members of the DW biofilm community and their associations have possibly developed over time in these environments. This could possibly support the idea of strong priority effects, where first colonizers will strongly determine the chronosuccession of events leading to establishment of successful biofilm structures in DWs.

### Conclusion.

In this study, we investigated diverse bacterial and fungal communities in biofilms formed on different DW rubber seals. Furthermore, abiotic conditions in DWs were shown to influence microbial community composition, and several putative microbial pathogens that are important to food safety and human health were presented. This study confirms that household appliances like dishwashers, colonized by polyextremotolerant bacteria and fungi, could present potential sources of domestically sourced infections. To understand and possibly prevent these phenomena, more studies should investigate fungal interactions with bacterial physiology and vice versa in biofilms formed on household appliances.

## MATERIALS AND METHODS

### Dishwasher sample information.

Microbial biofilms grown on the rubber seals of 24 different DWs in private dwellings across different Slovenian cities were sampled in this study (Table 1). The water supply at source of these DWs was characterized into soft or hard water based on an ion analysis method as previously performed (M. N. Babič, P. Zalar, and N. Gunde-Cimerman, presented at ISHAM, Guangzhou, China, 2013). Final concentrations were determined following the method from ISO Standard SIST EN ISO 11885:2009.

### Biofilm sampling and genomic DNA extraction.

Biofilm formed on up to 1 cm^2^ of the rubber seal surface (Fig. 1) was scraped off using a sterile scalpel, and the collected biomass was placed into a sterile tube and stored at −20°C until use. Genomic DNA extraction from 50 to 100 mg of biofilm biomass was performed using a MoBio Power Biofilm DNA isolation kit (Carlsbad, CA, USA) according to the manufacturer's instructions. Extraction controls were included during DNA extraction. The negative control contained ultrapure water (Milli-Q) in the same quantity as dishwasher biofilm biomass. The processing of the sample and the negative control was performed simultaneously and in the same way, according to the manufacturer's instructions. DNA concentrations were quantified for all samples using the Qubit dsDNA (double-stranded DNA) HS (high sensitivity) assay kit and by measuring the fluorescence on a Qubit fluorometer (Invitrogen, UK).

### 16S rRNA gene and nuclear ribosomal internal transcribed spacer (ITS) rRNA amplicon-based sequencing.

To determine the microbial diversity in biofilms associated with the selected DW rubber seals, amplicon sequencing based on the 16S rRNA gene and nuclear ribosomal internal transcribed spacer (ITS) region for bacterial and fungal diversity, respectively, was applied. The concentration of extracted DNA was quantified and adjusted to 5 ng/μl for all samples using ultrapure water (Milli-Q). For the PCR, 1 μl of the above prepared DNA was used. The variable regions V3 and V4 were used for bacterial identification by primers targeting the flanking conserved regions and amplified using the primers PRK341F (5′-CCT AYG GGR BGC ASCAG-3′) and MPRK806R (5′-GGATCTACNNGGTATSTAAT-3′) (69). The general eukaryote primers ITS7 (5′-GTGAATCATCGAATCTTTG-3′) (70) and ITS4 (5′-CAGACTTRTAYATGGTCCAG-3′) (71) were used to amplify the ITS2 region for sequencing. A blank was included as a negative control. PCR amplifications were done in two steps.

The first PCR amplification was done using PuRe *Taq* Ready-To-Go PCR Beads (GE Healthcare, United Kingdom) containing 1 μl of each primer. Bacterial PCR-I mix was amplified according to the following conditions: 94°C for 2 min, 35 cycles of 94°C for 20 s, 56°C for 20s, and 68°C for 30s, and final extension at 68°C for 5 min. Eukaryotic PCR-I amplifications were 94°C for 2 min, 35 cycles of 94°C for 30 s, 56°C for 30 s, and 72°C for 30 s, followed by 72°C for 5 min. The final products were then cooled on ice to minimize hybridization between specific PCR products and short nonspecific amplicons. Products were checked by running 5 μl of product on a 1.5% agarose gel. Sequencing primers and adaptors were added to the amplicon products in the second PCR step as follows: 2.0 μl 10× AccuPrime PCR buffer containing 15 mM MgCl_2_ (Invitrogen), 0.15 μl (MSM2) AccuPrime *Taq* DNA polymerase (2 units/μl; Life Technologies), 1.0 μl of each fusion primer, 2 μl of 10× diluted PCR product from first PCR, and water to a total of 20 μl reaction volume. The PCR-II conditions were as follows: 94°C for 2 min, followed by 15 cycles of 94°C for 30 s, 56°C for 30s, and 68°C for 30s, and final extension at 68°C for 5 min. Amplicons were size separated on a 1% agarose gel and purified using a Montage gel extraction kit (Millipore, Billerica, MA, USA). Amplicon concentrations were quantified for all samples using the Qubit dsDNA HS assay and by measuring the fluorescence on a Qubit fluorometer (Invitrogen, UK). Samples were sequenced using an Illumina MiSeq sequencer, employing paired-end reads, as described previously (72). Demultiplexing was performed by the MiSeq Control Software. Raw fastq files for both 16S rRNA gene and ITS data were processed with qiime_pipe, a wrapper around the QIIME (1.7) pipeline (available at https://github.com/maasha/qiime_pipe). The preprocess_illumina.rb script handles quality control, and for both data sets this was done using the same parameters. Merging of paired-end reads was done with a maximum of 20% mismatches and minimum length overlaps of 15 bp. Primers were identified with 2 maximum mismatches, and the amplicon was trimmed to only contain the sequence between the primers. Sequences were discarded if the average quality was less than 30. Chimera checks were performed with the QIIME script identify_chimeric_seqs.py using the usearch61 method. For 16S rRNA gene data, it was checked against GreenGenes (4 February 2011), and for ITS data, against UNITE (9 February 2014; dynamic version) databases. OTU picking was done at 97% for both data sets with QIIME's pick_otu.py script, and representatives of these OTUs were picked with the pick_rep_set.py script, both at default settings. For 16S rRNA gene data, trees were generated by alignment with the GreenGenes set using PyNast and FastTree through the QIIME wrappers. Representative sequences were classified through the Ribosomal Database Project (RDP) Classifier at the default 0.8 confidence threshold. The same databases were used for classification as for chimera checking.

### Microbial interaction network.

Positive correlations signifying cooccurrence and negative correlations signifying mutual exclusions were characterized by generating the Spearman cooccurrence network. The network based on normalized abundance was generated using the CoNet 1.0b6 plugin for Cytoscape 3.2.1, on the basis of the inbuilt nonparametric Spearman correlation coefficient with a minimal cutoff threshold of *r* ≥ ∣0.65∣ (*P* < 0.01, Bonferroni corrected) (73, 74). In this study, we present the correlation data for bacterial and fungal members that were coexisting in the same dishwashers (*n* = 18). OTUs with ≥50% sample representation were selected for the microbial network (*n* ≥ 9).

### Data analysis.

Alpha diversity analyses were performed using PAST software ver.2.17 (75). Alpha diversity indices were calculated on OTU counts rarefied to 4,000 counts per sample for bacterial sequences, and samples below 4,000 bacterial counts were not included in this analysis. Fungal counts were rarefied to 400 counts per sample for this particular analysis. Fungal sequences rarefied to the lowest sequencing depth allowed DW samples with replicate conditions to be maintained. The following indices were used to assess the diversity: sample richness, Shannon (H), and Chao-1. The effect of DW conditions on alpha diversity indices was statistically assessed using the Wilcoxon-Mann-Whitney *t* test (*P* < 0.05).

Multivariate beta diversity analyses were done using nonrarefied counts (76). As the contingency tables featured 1,000-fold variation in abundance, a log_10_ transformation was applied in order to have all the information possible and to satisfy distributional assumptions. The transformed compositional data set was subjected to a redundancy analysis (RDA) using DW conditions as factors. The significance of the model and of the RDA axes and factors were estimated by ANOVA using Euclidean distances and 999 permutations, (significance, *P* < 0.05). In addition, PERMANOVA using 999 permutations and the Bray-Curtis dissimilarity index was performed to assess the significance of DW conditions. Selections of taxa (phylum and genus levels) with significant changes in prevalence grouped by DW conditions were performed in STAMP 2.1.3 (77) using multiple and group comparisons and significance checked using inbuilt ANOVA (Tukey-Kramer *post hoc* tests) and Welch's *t* test (Bonferroni corrected). The selected significant taxa were plotted as a heatmap using dissimilarity indices computed based on Euclidean distances, average clustering, and scaled counts. The heat maps were clustered based on CONISS cluster analysis. RDA, PERMANOVA, and principal component (PC) plots, and heatmaps were generated using various R packages, namely, gplots, vegan, rioja and RColorBrewer available for RGui 3.2.0 (78).

### Accession number(s).

The sequence data sets generated and analyzed during the current study are available at the NCBI Sequence Read Archive (SRA) under the BioProject accession numbers PRJNA315977 for bacterial reads and PRJNA317625 for fungal reads.

## Supplementary Material

Supplemental material
