# Supplementary material for: Microbial Diversity and Putative Opportunistic Pathogens in Dishwasher Biofilm Communities
Source: Appl Environ Microbiol. 2018 Feb 14;84(5):e02755-17. doi: 10.1128/AEM.02755-17 (PMC5812945; doi:10.1128/AEM.02755-17)
Supplement: Supplemental material [file supp_84_5_e02755-17__index.html]

Supplemental material 

# Microbial Diversity and Putative Opportunistic Pathogens in Dishwasher Biofilm Communities

## Supplemental material

- Supplemental file 1 -

  Microbial composition in individual samples at the phylum level (Fig. S1); significant cooccurrence and coexclusion interactions (Fig. S2); 16S rRNA gene- and ITS gene-based amplicon profiles (Table S1); dishwasher alpha diversity summary after rarefaction of sequence counts (Table S2); *P* values from pairwise comparisons (Tables S3 and S4); summary from redundancy-based ANOVA (Table S5); PERMANOVA (Table S6).

  PDF, 623K
