# Supplementary material for: Microbial Diversity and Putative Opportunistic Pathogens in Dishwasher Biofilm Communities
Source: Appl Environ Microbiol. 2018 Feb 14;84(5):e02755-17. doi: 10.1128/AEM.02755-17 (PMC5812945; doi:10.1128/AEM.02755-17)
Supplement: Supplemental material [file AEM.02755-17_zam005188350s1.pdf]

Supplementary information

Supplementary Figure S1: Microbial composition in individual samples at the phylum level. (A) Fungal and (B) bacterial abundance (%) across DW samples. The low abundant phyla Acidobacteria, Chloroflexi, Cyanobacteria, Spirochaetes, Synergistetes, Verrucomicrobia and other unclassified/unassigned reads are combined in the miniscule 'others'. Gap in the dataset refer to samples where sequence data could not be generated.

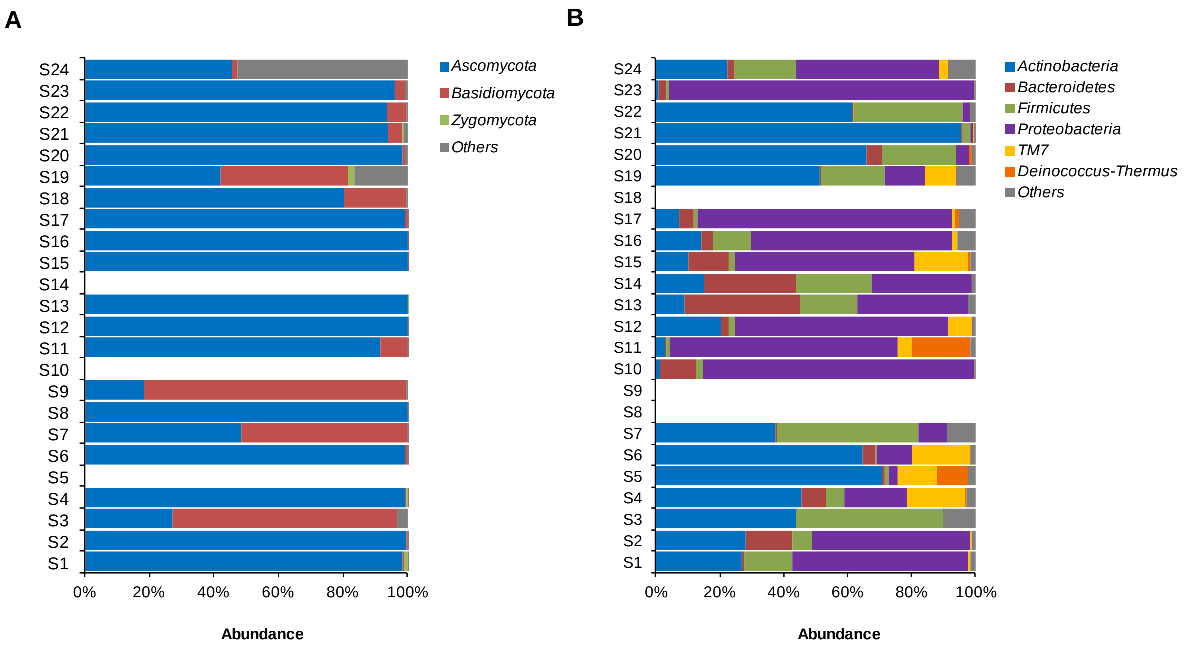

Supplementary Figure S2: Significant co-occurrence and co-exclusion interactions among bacterial and fungal OTUs in the DWs microbiome. Predicted pairwise interaction network between the bacterial and fungal OTUs generated from the OTU matrix. The displayed pairwise co-occurrences appeared in at least 50% of all samples and were dominated by positive correlations.

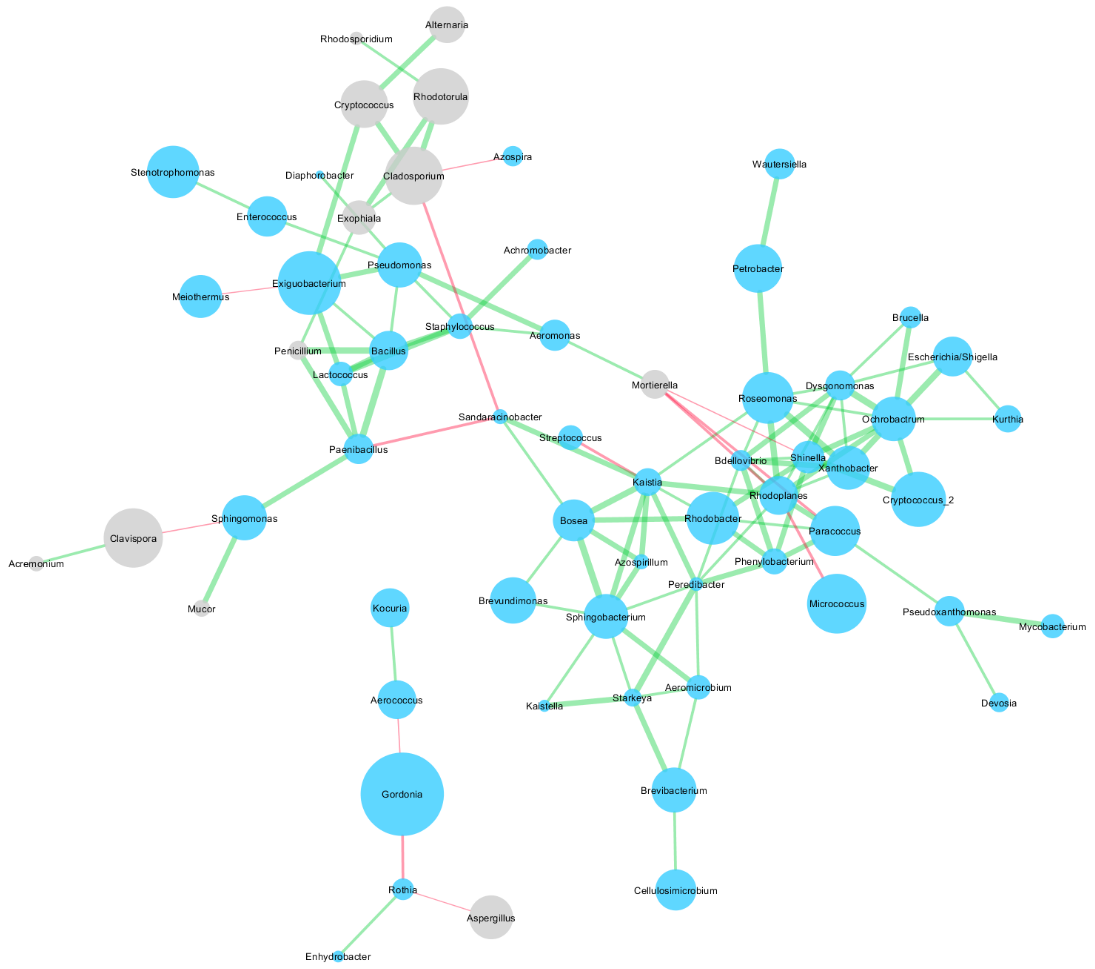

Supplementary Table S1: Summary table of 16S rRNA gene and ITS gene based amplicon profiles obtained. **'**No reads' indicates samples where sequences reads could not be generated. SRR represents the SRA accession numbers obtained after deposition of raw reads to NCBI database.

	**Bacterial Richness**				**Fungal Richness**		
**Samples**	OTUs	Counts	SRR	Samples	OTUs	Counts	SRR
**S1**	97	12056	*3335213*	**S1**	26	4536	*3354512*
**S2**	104	14880	*3279031*	**S2**	30	47413	*3354513*
**S3**	35	13294	*3335242*	**S3**	64	52407	*3354514*
**S4**	105	11086	*3343755*	**S4**	32	53664	*3354515*
**S5**	56	4812	*3343756*	**S5**	No reads	No reads	No reads
**S6**	104	8872	*3343757*	**S6**	43	9514	*3354535*
**S7**	112	5790	*3343758*	**S7**	26	28574	*3354573*
**S8**	No reads	No reads	No reads	**S8**	22	37174	*3354574*
**S9**	No reads	No reads	No reads	**S9**	60	24041	*3354575*
**S10**	115	17912	*3343759*	**S10**	No reads	No reads	No reads
**S11**	55	1511	*3343760*	**S11**	33	14060	*3354576*
**S12**	60	961	*3343761*	**S12**	26	19847	*3354577*
**S13**	104	14995	*3343763*	**S13**	28	9602	*3354578*
**S14**	96	6661	*3343796*	**S14**	No reads	No reads	No reads
**S15**	107	9683	*3343797*	**S15**	15	10340	*3354579*
**S16**	50	127	*3343798*	**S16**	20	3520	*3354580*
**S17**	107	3580	*3343799*	**S17**	18	3601	*3354581*
**S18**	No reads	No reads	No reads	**S18**	30	2162	*3354582*
**S19**	95	24429	*3343800*	**S19**	104	4372	*3354583*
**S20**	29	100	*3343802*	**S20**	25	660	*3354584*
**S21**	71	8529	*3343803*	**S21**	45	891	*3354585*
**S22**	110	26115	*3343805*	**S22**	27	472	*3354587*
**S23**	92	15960	*3343810*	**S23**	30	436	*3354588*
**S24**	150	13036	*3335236*	**S24**	40	49511	*3354600*

Supplementary Table S2: Dishwasher alpha diversity summary post rarefaction of sequence counts. 'NA' indicates samples in which sequences reads were not generated.

**Bacterial Diversity**				**Fungal Diversity**			
**Samples**	*Richness Taxa_S*	*Shannon_H*	*Chao-1*	**Samples**	Richness Taxa_S	Shannon_H	Chao-1
**S1**	69	2.7	78	**S1**	10	0.7633	11
**S2**	75	2.9	82	**S2**	5	0.32	5
**S3**	18	1.2	19	**S3**	16	1.658	16
**S4**	75	2.2	103	**S4**	10	0.9061	12
**S5**	48	1.7	54	**S5**	NA	NA	NA
**S6**	72	2.0	83	**S6**	13	1.441	16
**S7**	87	2.7	108	**S7**	3	0.03494	4
**S8**	NA	NA	NA	**S8**	19	1.601	24
**S9**	NA	NA	NA	**S9**	NA	NA	NA
**S10**	78	2.4	92	**S10**	15	1.655	17
**S11**	-	-	-	**S11**	7	0.3897	9
**S12**	-	-	-	**S12**	8	0.8721	8
**S13**	68	2.5	79	**S13**	NA	NA	NA
**S14**	73	2.8	103	**S14**	4	0.697	4
**S15**	84	3.0	105	**S15**	10	1.039	25
**S16**	-	-	-	**S16**	7	0.261	10
**S17**	-	-	-	**S17**	12	1.632	14
**S18**	NA	NA	NA	**S18**	42	2.714	46
**S19**	51	2.2	62	**S19**	14	0.8477	16
**S20**	-	-	-	**S20**	26	1.481	32
**S21**	50	2.0	55	**S21**	21	2.483	23
**S22**	59	2.2	74	**S22**	26	1.276	30
**S23**	57	1.3	83	**S23**	22	1.669	31
**S24**	110	3.6	133	**S24**	10	0.7633	11

Supplementary Table S3: Summary of p-values from pairwise comparisons on bacterial alpha diversity indices using Wilcoxon-Mann-Whitney.

Chao-1	**Year**	**0-4**	**5-7**	**Freq**	**1-3**	**7**	**WH**	**MS**	**SH**	**MH**
	**0-4**	-	0.43	**1-3**	-	0.56	**MS**	-	0.55	0.31
	**5-7**	0.43	-	**7**	0.56	-	**SH**	0.55	-	0.62
	**8**	0.47	0.21	**14**	0.99	0.70	**MH**	0.31	0.62	-
							**H**	0.27	0.49	0.72
Shannon_H	**Year**	**0-4**	**5-7**	**Freq**	**1-3**	**7**	**WH**	**MS**	**SH**	**MH**
	**0-4**	-	0.52	**1-3**	-	0.16	**MS**	-	0.35	0.07
	**5-7**	0.52	-	**7**	0.16	-	**SH**	0.35	-	0.28
	**8**	0.37	0.22	**14**	0.63	0.16	**MH**	0.07	0.28	-
							**H**	0.28	0.71	0.66
Richness	**Year**	**0-4**	**5-7**	**Freq**	**1-3**	**7**	**WH**	**MS**	**SH**	**MH**
	**0-4**	-	0.41	**1-3**	-	0.28	**MS**	-	0.42	0.24
	**5-7**	0.41	-	**7**	0.28	-	**SH**	0.42	-	0.67
	**8**	0.30	0.13	**14**	0.99	0.46	**MH**	0.24	0.67	-
							**H**	0.20	0.46	0.65

Supplementary Table S4: Summary of p-values from pairwise comparisons on fungal alpha diversity indices using Wilcoxon-Mann-Whitney (* $p \leq 0.05$)

Chao-1	**Year**	**0-4**	**5-7**	**Freq**	**0-6**	**WH**	**MS**	**SH**	**MH**
	**0-4**	-	0.72	**7-14**	0.20	**MS**	-	0.19	0.27
	**5-7**	0.72	-			**SH**	0.19	-	0.71
	**8**	0.91	0.82			**MH**	0.27	0.71	-
						**H**	0.19	0.01*	0.02*
Shannon_H	**Year**	**0-4**	**5-7**	**Freq**	**0-6**	**WH**	**MS**	**SH**	**MH**
	**0-4**	-	0.04*	**7-14**	0.31	**MS**	-	0.28	0.64
	**5-7**	0.04*	-			**SH**	0.28	-	0.38
	**8**	0.84				**MH**	0.64	0.38	-
						**H**	0.02*	0.0009*	0.003*
Richness	**Year**	**0-4**	**5-7**	**Freq**	**0-6**	**WH**	**MS**	**SH**	**MH**
	**0-4**	-	0.56	**7-14**	0.4	**MS**	-	0.33	0.25
	**5-7**	0.56	-			**SH**	0.33	-	0.85
	**8**	0.98	0.60			**MH**	0.25	0.85	-
						**H**	0.05*	0.01*	0.01*

Supplementary Table S5: Summary from redundancy based ANOVA analysis done using each of the factors (999 permutations). The composition of the bacterial community was significantly affected by the factors 'years of use' and 'frequency' of DWs. The composition of fungal community was significantly affected by years of use, frequency of use and tap water hardness. df: degrees of freedom; F: ratio of the between groups variance and within groups variance; p: p-values. Star indicates the level of significance of each factor: '*' $p < 0.05$.

Source	Factors	df	Variance (%)	F	p
Bacteria Diversity	Years (0-4y, 5-7y and 8y)	2	23.44	1.34	0.047*
	Temperature (≥65°C, ≤60°C)	1	9.63	1.11	0.299
	Frequency of use (1-3, 7 and 14)	2	27.21	1.55	0.022*
	Water Hardness (H,MH,SH and MS)	3	24.00	0.91	0.660
Fungi Diversity	Years (0-4y, 5-7y and 8y)	2	9.47	1.32	0.043 *
	Temperature (≥65°C, ≤60°C)	1	4.04	1.12	0.280
	Frequency of use (0-7 and 14)	1	5.78	1.61	0.034 *
	Water Hardness (H,MH,SH and MS)	3	18.87	1.76	0.017 *

Supplementary Table S6: PERMANOVA based on Bray-Curtis dissimilarity index and dishwasher conditions using 999 permutations. The analyses were executed using the vegan package for R software. df: degrees of freedom; R^2: coefficient of determination; p: p-values. Star indicates the level of significance of each factor: '*' $p < 0.05$.

Source	Factors	df	R^2	p
Bacteria Diversity	Years of use	2	0.11	0.186
	Temperature	1	0.05	0.386
	Water hardness	3	0.13	0.519
	Frequency of use	2	0.14	0.045*
	Residuals	12	0.56	
Fungi Diversity	Years of use	2	0.10	0.200
	Temperature	1	0.06	0.135
	Water Hardness	3	0.17	0.018*
	Frequency of use	1	0.09	0.127
	Residuals	13	0.58	
